# Selective Coordination of Cu^2+^ and Subsequent Anion Detection Based on a Naphthalimide-Triazine-(DPA)_2_ Chemosensor

**DOI:** 10.3390/bios10090129

**Published:** 2020-09-22

**Authors:** Artur J. Moro, Miguel Santos, Mani Outis, Pedro Mateus, Pedro M. Pereira

**Affiliations:** 1 LAQV-REQUIMTE, Departamento de Química, CQFB, Universidade Nova de Lisboa, 2829-516 Caparica, Portugal; miguelmsantos@fct.unl.pt (M.S.); m.hosseinzadeh@campus.fct.unl.pt (M.O.); pm.mateus@fct.unl.pt (P.M.); 2 Bacterial Cell Biology, MOSTMICRO, Instituto de Tecnologia Química e Biológica António Xavier, Universidade Nova de Lisboa, 2780-157 Oeiras, Portugal; pmatos@itqb.unl.pt

**Keywords:** fluorescent chemosensor, naphthalimide, copper (II), phosphate derivatives, super-resolution fluorescence microscopy

## Abstract

A new fluorescent chemosensor for copper (II) and subsequent anion sensing was designed and fully characterized. The sensor consisted of a 1,8-naphthalimide core, bearing two terminal dipicolylamine (DPA) receptor units for binding metal cations, and an ethoxyethanol moiety for enhanced water solubility. The DPA units are connected to position 4 of the fluorophore via a triazine-ethylenediamine spacer. Fluorescence titration studies of the chemosensor revealed a high selectivity for Cu^2+^ over other divalent ions, the emissions were strongly quenched upon binding, and a stability constant of 5.52 log units was obtained. Given the distance from DPA chelating units and the fluorophore, quenching from the Cu^2+^ complexation suggests an electron transfer or an electronic energy transfer mechanism. Furthermore, the Cu^2+^-sensor complex proved to be capable of sensing anionic phosphate derivatives through the displacement of the Cu^2+^ cation, which translated into a full recovery of the luminescence from the naphthalimide. Super-resolution fluorescence microscopy studies performed in HeLa cells showed there was a high intracellular uptake of the chemosensor. Incubation in Cu^2+^ spiked media revealed a strong fluorescent signal from mitochondria and cell membranes, which is consistent with a high concentration of ATP at these intracellular sites.

## 1. Introduction

The field of chemosensors has experienced a “boom”, mainly due to the appearance of coordination compounds bearing fluorophores, which correlate their luminescence to the amount of cation bound to the chelating/receptor unit.

Over the past few decades, a multitude of chemosensor systems that are capable of detecting metal cations and anions have been developed for biological applications. As such, a wide range of examples of chemosensors for sensing ions can be found in the literature [1,2,3,4].

**Figure 1:**
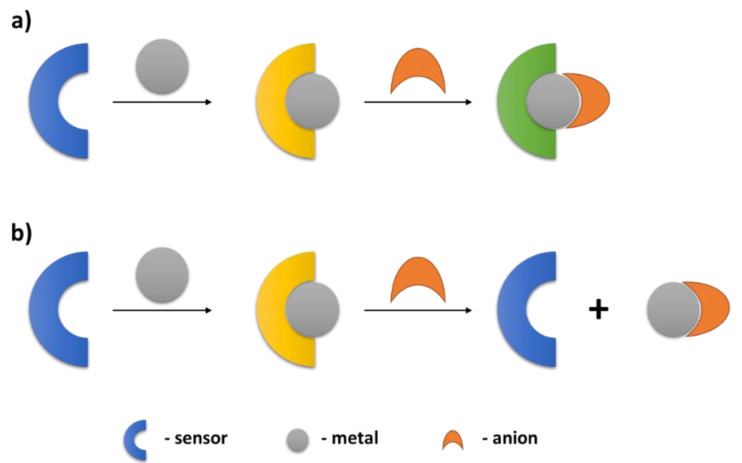
Two approaches for anion sensing: (**a**) sequential; and (**b**) displacement.

A currently employed strategy is the use of chelating moieties coupled with fluorophores that are capable of coordinating metal cations, which are subsequently used to bind anionic species in a sequential manner, mainly by taking advantage of the strength of the coordination bond (Figure 1a). In particular, the scientific community has focused on detecting phosphate derivatives because of their relevant role in biologic systems, especially in the regulation of metabolic mechanisms in cells [5]. There are many examples of this, including the use of cyclam [6,7], cyclen [8,9,10] and other polyamines [11,12,13,14]. One of the most successful approaches was developed by Hamachi et al. It involves coupling fluorophores to a di-(2-picolyl) amine unit, DPA, which has a particularly high affinity for Zn^2+^, Cd^2+^ and Cu^2+^, and then using the fluorophore-DPA-metal complex as a luminescent sensor for phosphate species [15,16,17]. Although many other successful examples have been reported, the vast majority of these systems involve the use of a high percentage of organic co-solvents to ensure the full solubility of the chemosensors. Therefore, such sensor systems are not entirely adequate for application in the field of biology.

An alternative approach (the displacement approach) consists of a coordination complex in which the presence of anionic species revives the non-coordinated spectroscopic behavior of the sensor [18]. In these systems, the ligand must be able to coordinate a metal ion that changes its initial optical properties, but this coordination should be relatively weak so that an anionic analyte can easily displace the metal to restore the original spectroscopic features of the ligand (Figure 1b).

We have previously reported a naphthalimide-DPA chemosensor capable of binding to Zn^2+^, which was subsequently used to detect the presence of ATP with some selectivity over other phosphate derivatives [19]. The sensing mechanism was based on photoinduced electron transfer from the aliphatic amine of the DPA moiety, which was influenced by metal coordination and its posterior association to ATP. This sensor was later appended to the surface of silica nanoparticles for in vitro studies in cells [20].

In this work, we designed a new 1,8-naphthalimide derivative coupled to an ethylenediamine-triazine unit with two appended DPA chelating moieties. Triazine exerts a strong electron-withdrawing effect on the lone electron pair of the nitrogen from the ethylenediamine spacer. Consequently, this free electron pair no longer has an effect on naphthalimide emission via photoinduced electron transfer (PET) [21]. Moreover, this electron-withdrawing effect is expected to render the DPA units weakly coordinating, such that the metal ion can be then be displaced by an anionic analyte.

## 2. Materials and Methods

All solvents used in the reactions were of analytical grade and purchased from Sigma-Aldrich (St. Louis, MO, USA). All reagents were purchased from Sigma-Aldrich and used as such without further purification.

### 2.1. Synthesis

Synthesis of 6-chloro-N^2^,N^2^,N^4^,N^4^-tetrakis(pyridin-2-ylmethyl)-1,3,5-triazine-2,4-diamine (**1**)

A solution of di-(2-picolyl)amine (DPA, 1 g, 5 mmol) and diisopropylethylamine (DIPEA, 1.2 eqs., 0.78 g, 6 mmol) in 20 mL of DCM was added dropwise to a solution of cyanuric chloride (0.92 g, 5 mmol) in 40 mL of dichloromethane (DCM) in an ice bath. The mixture was kept under ice and followed by TLC until the cyanuric chloride (~30 min) was fully consumed. At this point, an equal portion of the DPA/DIPEA solution was further added dropwise, after which the ice bath was removed, and the reaction was allowed to warm to room temperature and stirred overnight. The reaction mixture was then washed with 50 mL saturated NaHCO3 solution, two times with 50 mL of water and once with 50 mL of brine. Afterwards, the organic extract was dried with Na_2_SO_4_, filtered and the volatiles were evaporated and the residue was purified by flash chromatography (SiO_2_, DCM/MeOH 96:4, R_f_ = 0.37), yielding 2.13 g (η = 83.5%) of pure **1**. ^1^H NMR (400 MHz, CDCl_3_) δ 8.52 (d, *J* = 4.2 Hz, 1H), 8.41 (d, *J* = 4.2 Hz, 1H), 7.64 (t, *J* = 7.6 Hz, 1H), 7.38 (t, *J* = 7.7 Hz, 1H), 7.31 (d, *J* = 7.8 Hz, 1H), 7.20–7.13 (m, 1H), 7.07–7.01 (m, 1H), 6.90 (d, *J* = 7.8 Hz, 1H), 5.04 (s, 2H), 4.83 (s, 2H). ^13^C NMR (101 MHz, CDCl_3_) δ 169.87, 165.74, 157.23, 157.19, 149.31, 149.16, 136.74, 136.42, 122.42, 122.40, 121.94, 121.24, 52.09, 51.97. ESI-MS *m*/*z* 510.19 [M + H]^+^.

Synthesis of 6-bromo-2-(2-(2-hydroxyethoxy)ethyl)-1H-benzo[de]isoquinoline-1,3(2H)-dione (**2**)

Both 4-bromo-1,8-naphthalic anhydride (3 g, 10.8 mmol) and 2-(2-aminoethoxy)ethanol (1.15 g, 10.9 mmol) were refluxed in 100 mL of ethanol in a 250 mL round bottom flask. The reaction was complete when the initially milky suspension becomes a brown clear solution (~2 h). At this point, stirring was halted and the reaction was left standing overnight, resulting in the formation of pale-yellow crystals of the product. The crystals were filtered and washed with cold ethanol. In the end, 3.38 g of **2** was obtained (η = 86%). ^1^H NMR (400 MHz, CDCl_3_) δ 8.66 (d, *J* = 7.2 Hz, 1H), 8.57 (d, *J* = 8.5 Hz, 1H), 8.41 (d, *J* = 7.8 Hz, 1H), 8.04 (d, *J* = 7.8 Hz, 1H), 7.85 (t, *J* = 7.9 Hz, 1H), 4.44 (t, *J* = 5.6 Hz, 2H), 3.85 (t, *J* = 5.5 Hz, 2H), 3.73–3.62 (m, 4H). ^13^C NMR (101 MHz, CDCl_3_) δ 163.96, 163.94, 133.50, 132.29, 131.46, 131.17, 130.68, 130.54, 129.08, 128.14, 122.93, 122.06, 72.26, 68.36, 61.85, 39.66.

Synthesis of 6-((2-aminoethyl)amino)-2-(2-(2-hydroxyethoxy)ethyl)-1H-benzo[de]isoquinoline-1,3(2H)-dione (**3**)

In a 50 mL round-bottom flask, **2** (0.5 g, 1.4 mmol) and ethylenediamine (0.1 g, 1.2 eqs., 1.7 mmol) were dissolved in 10 mL of 2-methoxyethanol and the mixture was refluxed overnight. TLC (DCM:MeOH 9:1) revealed the full consumption of **2** and the formation of a strong-yellow luminescent product. The volatiles were evaporated and the resulting residue was purified by silica-gel flash chromatography, starting at DCM:MeOH 9:1 and increasing polarity up to 3:1, yielding 0.27 g of **3** (η = 57%). ^1^H NMR (400 MHz, CD_3_OD) δ 8.55 (d, *J* = 8.5 Hz, 1H), 8.51 (d, *J* = 7.3 Hz, 1H), 8.37 (d, *J* = 8.5 Hz, 1H), 7.66 (t, *J* = 7.9 Hz, 1H), 6.86 (d, *J* = 8.6 Hz, 1H), 4.38 (t, *J* = 6.1 Hz, 2H), 3.80 (t, *J* = 6.1 Hz, 2H), 3.63 (dd, *J* = 7.9, 3.4 Hz, 3H), 3.58 (t, *J* = 6.4 Hz, 2H), 3.07 (t, *J* = 6.3 Hz, 2H). ^13^C NMR (101 MHz, DMSO-d_6_) δ 164.26, 163.43, 150.74, 134.62, 131.32, 129.79, 124.91, 122.19, 120.87, 108.92, 104.48, 72.54, 67.54, 60.64, 49.05, 37.76.

Synthesis of 6-((2-((4,6-bis(bis(pyridin-2-ylmethyl)amino)-1,3,5-triazin-2-yl)amino)ethyl)amino)-2- (2-(2-hydroxyethoxy)ethyl)-1H-benzo[de]isoquinoline-1,3(2H)-dione (**4**)

In a 25 mL round-bottom flask, **1** (0.363 mg, 0.71 mmol) and **3** (0.245 mg, 0.71 mmol) were dissolved in 20 mL of acetonitrile. A solution of K2CO3 (2 eqs. 197 mg, 1.42 mmol) in 4 mL of water was added and the reaction was refluxed for 24 h. The reaction mixture was then cooled to room temperature, filtered, and concentrated in a rotatory evaporator. The residue was purified by silica-gel flash chromatography (DCM:MeOH 20:1, R_f_ = 0.3), yielding 143 mg of pure **4** (η = 24%). ^1^H NMR (400 MHz, DMSO-d_6_) δ 8.55 (d, *J* = 8.5 Hz, 1H), 8.48–8.33 (m, 5H), 8.11 (d, *J* = 8.5 Hz, 1H), 7.73–7.57 (m, 4H), 7.48 (m, 2H), 7.22 (dd, *J* = 12.8, 7.3 Hz, 4H), 7.17–7.12 (m, 2H), 7.04 (t, *J* = 5.7 Hz, 1H), 6.92 (d, *J* = 7.7 Hz, 1H), 6.87 (d, *J* = 7.8 Hz, 1H), 6.63 (d, *J* = 8.7 Hz, 1H), 4.85 (s, 4H), 4.76 (s, 2H), 4.71 (s, 2H), 4.68 (s, 1H), 4.19 (t, *J* = 6.4 Hz, 2H), 3.61 * (2H), 3.45 (d, *J* = 1.6 Hz, 8H), 3.36 (d, *J* = 5.4 Hz, 3H). ^13^C NMR (126 MHz, DMSO-d_6_) δ 166.37, 165.76, 164.32, 163.40, 158.91, 158.73, 158.58, 151.17, 149.48, 149.43, 149.18, 137.20, 137.13, 136.91, 134.68, 131.26, 129.87, 128.97, 124.74, 122.58, 122.42, 122.14, 121.85, 121.68, 121.61, 120.50, 107.95, 104.19, 72.48, 67.56, 60.64, 52.23, 52.15, 51.79, 51.59, 43.03, 38.96, 38.85. HR-ES-MS (+) *m*/*z*: 817.3680 ([M + H]^+^, cacld.: 817.3681).

* This signal was assigned with COSY NMR, since the peak is overlapped with that of water.

### 2.2. UV-Vis and Fluorescence Titrations

Solutions for absorption and fluorescence measurements were prepared by adding an aliquot of 30 µL of a methanolic solution of **4**, to 2970 µL of aqueous buffer. For all metal and anion titrations, 10 mM HEPES buffer at pH 7.0 ± 0.2 was used. Titrations were performed by adding a solution containing both metal and **4** to a cuvette containing solely **4**, to ensure that the concentration of the latter remained constant.

Absorption spectra were acquired in a 1 cm quartz cuvette on a Varian Cary 100 Bio UV- spectrophotometer. Emission spectra were obtained in a 1 cm fluorescence quartz cuvette, using a Horiba-Jobin-Yvon SPEX Fluorolog 3.22 spectrofluorimeter. Fluorescence quantum yield for **4** was determined using Acridine Yellow G as a reference (ϕ_f_ = 0.57, in methanol) [22]. The binding constant for **4**-Cu^2+^ was determined by fitting the experimental data to a Henderson-Hasselbalch binding model using the Solver Add-In from Microsoft Excel [23]. The limit of detection (LOD) of Cu^2+^ was determined by measuring five independently prepared samples over a range of concentrations with a linear variation in the fluorescent emission, and applying the formula: LOD = 3σ/K, where *σ* represents the standard deviation, and *K* represents the slope over the linear range.

### 2.3. Cell Viability and Fluorescence Microscopy Studies

For biocompatibility studies, HeLa cells were seeded at 0.05 × 106 and incubated in DMEM containing 10% FBS, 100 U mL^−1^ penicillin and 100 μg mL^−1^ streptomycin at 37 °C with 5% CO_2_ in a humidified incubator in the presence or absence of Cu^2+^ with increasing concentrations of chemosensor **4** (from 0 to 12 micromolar). Cell viability was determined by cell counting and live dead stain (ThermoFisher, Waltham, MA, USA) using a fluorescence cell drop (DeNovix, Wilmington, DE, USA), both following the manufacturer’s recommendations.

For microscopy experiments, HeLa cells were grown in DMEM containing 10% FBS, 100 U mL^−1^ penicillin and 100 μg mL^−1^ streptomycin at 37 °C with 5% CO_2_ in a humidified incubator. Before imaging cells were seeded at 0.3 × 106 on to #1.5 high performance 25 mm glass coverslips and grown for 24 h. For imaging, the cells were washed 1X with DMEM, the wash media was replaced with DMEM with 10 μg/mL **4** and 5 μg/mL FM5-95 membrane dye (Molecular Probes, Eugene, OR, USA), with or without 25 μM CuSO4, and cells were incubated for 5 min at 37 °C with 5% CO_2_ in a humidified incubator. Before imaging, the cells were washed 2X with DMEM and coverslips were mounted on an ATTOfluor Cell chamber (ThermoFisher) or by using a parafilm slide as previously described [24].

Super-resolution structured illumination microscopy (SIM) imaging was performed using an Elyra PS.1 microscope (Zeiss, Oberkochen, Germany) with a Plan-Apochromat 63×/1.4 oil DIC M27 objective. SIM images were acquired using three grid rotations with 34 μm grating period for the 561 nm laser, 28 μm period for 488 nm laser. Images were captured using a Pco.edge 5.5 camera and reconstructed using ZEN software (black edition, 2012, version 8.1.0.484) based on a structured illumination algorithm, using synthetic, channel-specific optical transfer functions and noise filter settings ranging from −6 to −8.

## 3. Results and Discussion

### 3.1. Synthesis

Chemosensor **4** was synthesized using a convergent synthesis, based on previously reported procedures. Briefly, two equivalents of di-(2-picolyl)amine were sequentially added to cyanuric chloride at 0 °C, to yield 6-chloro-N^2^,N^2^,N^4^,N^4^-tetrakis(pyridin-2-ylmethyl)-1,3,5-triazine-2,4-diamine (**1**) [25], to form our metal chelating moiety with two coordination centers.

For the synthesis of the fluorophore, we took advantage of the chemical versatility of the central 1,8-naphthlic core, which allows for facile introduction of any terminal amine. In this case, we chose to introduce an ethoxyethanol tail in order to increase the overall water solubility of the final molecule, by reacting 4-bromo-1,8-naphthalic anhydride with 2-(2-aminoethoxy)ethanol to afford 6-bromo-2-(2-(2-hydroxyethoxy)ethyl)-1H-benzo[de]isoquinoline-1,3(2H)-dione (**2**).

**Scheme 1:**
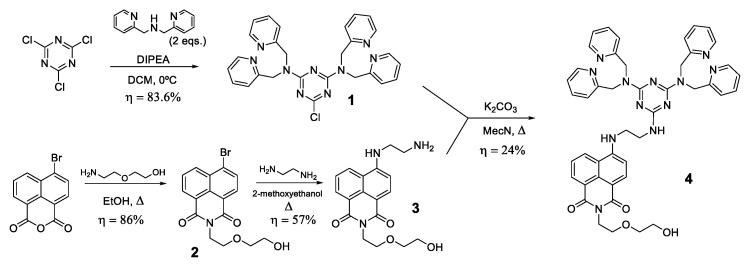
Synthetic route to chemosensor **4**.

Nucleophilic aromatic substitution of the bromine with ethylenediamine yielded 6-((2-aminoethyl)amino)-2-(2-(2-hydroxyethoxy)ethyl)-1H-benzo[de]isoquinoline-1,3(2H)-dione (**3**), a 4-amino-1,8-naphthalimide, which is already a highly luminescent fluorophore. Combining **1** and **3** in basic conditions yielded the final chemosensor molecule **4**. The full synthetic scheme is shown in Scheme 1.

### 3.2. UV-Vis and Fluorescence Studies

UV-Vis spectra of **4** in HEPES buffer indicated a single absorption band in the visible region, with maximum at ca. 460 nm. Molar absorptivity at this wavelength was found to be 12,082 (±172) M^−1^ cm^−1^, typical for 4-amino-1,8-naphthalimide dyes. Emission spectra exhibited a maximum fluorescence intensity at ca. 550 nm, with a fluorescence quantum yield of 0.19.

**Figure 2:**
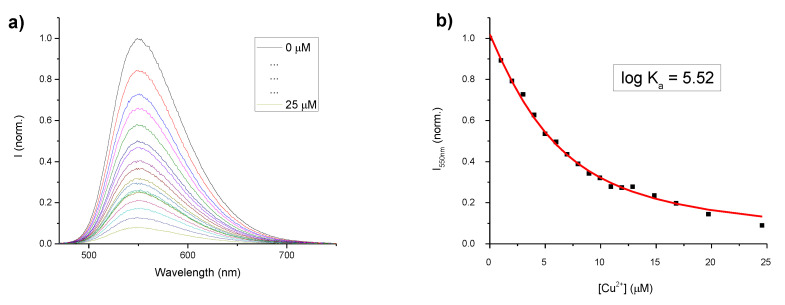
(**a**) Emission spectra from **4** in the presence of increasing amounts of Cu^2+^; (**b**) Normalized emission changes in **4** upon addition of Cu^2+^ (black dots) and respective exponential fitting (red line). Conditions: [4] = 5.0 µM; pH 7.0 ± 0.2 buffered with 10 mM HEPES; T = 298 K; λ_exc_ = 458 nm.

Titrations with several divalent cations were performed and followed by UV-Vis and fluorescence spectroscopies. UV-Vis spectra of **4** suffer a slight decrease in absorption (10–20%) upon metal addition, a behavior common to all studied cations (Figure S1). The emission spectra revealed a more interesting profile, with the sensor exhibiting a strong luminescence quenching with Cu^2+^ while no significant changes in the spectra were observed for any other cation (Figure 2a and Figure S2), indicating a high selectivity for this particular cation. The limit of detection for Cu^2+^ was found to be 2.09 µM, with the quantum yield for **4**-Cu^2+^ calculated at 0.015. The fact that no changes were observed in the absorption spectra suggests that emission of **4** is quenched upon copper(II) binding due to an electron transfer or an electronic energy transfer mechanism involving the transition metal and the nearby excited naphthalimide fluorophore [26,27,28].

The plot of total emission from **4** against the concentration of Cu^2+^ fits well to a 1:1 binding model, and results in an apparent stability constant of 5.52 log units (Figure 2b). The Cu^2+^ cation is expected to be coordinated by the nitrogen atoms of the two pyridyl groups of one of the DPA moyeties and by the nitrogen atom of the respective tertiary amine, with the remaining coordination sites being occupied by one or two water molecules (Figure S3). The apparent stability constant value that was determined is much lower than that found for the DPA ligand alone (log K = 13.85) [29], which can be ascribed to the strong electron-withdrawing effect exerted by the triazine core on the lone electron pair of the nitrogen from the DPA unit, rendering the whole chelating moiety weakly coordinating. Refining the fit to a 2:1 binding model produces very little change in the global fitting, with the first association constant (from free **4** to (**4**-Cu)^2+^) being essentially the same as that calculated for a 1:1 binding model (Figure S4). This means that coordination of a second Cu^2+^ is not favored due to electrostatic repulsions arising from the close proximity of the first coordinated Cu^2+^ cation, and again, because of the weak coordinating ability of the triazine-DPA ensemble.

**Figure 3:**
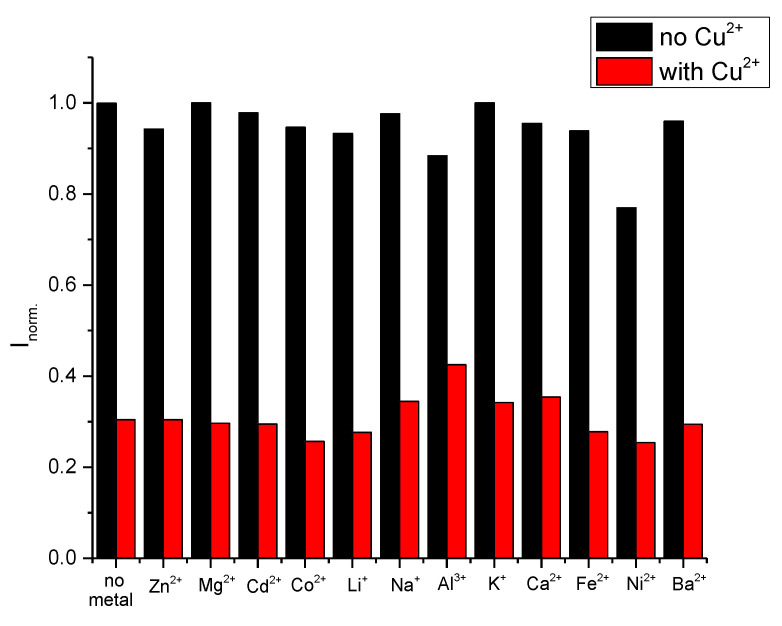
Competitive binding of **4** to 2 equivalents of Cu^2+^, in the presence of 5 equivalents of other divalent metal ions.

Competitive binding assays were also performed in the presence of other divalent metal ions (Figure 3). Changes in the emission of **4** before and after the addition of Cu^2+^ were essentially unaffected, even with the excess of other metals.

**Figure 4:**
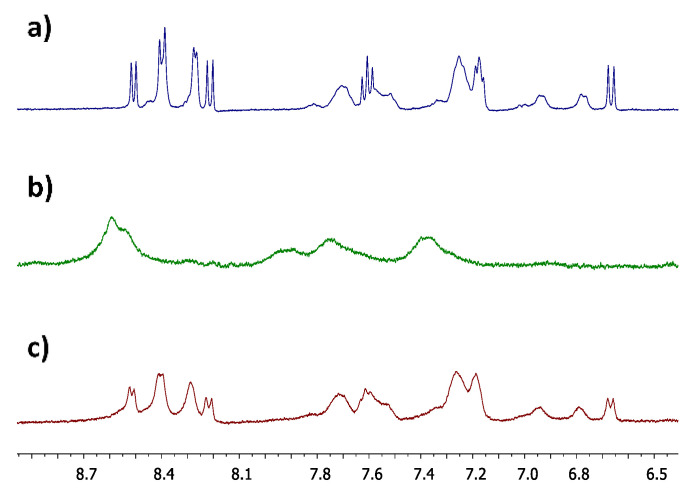
Aromatic region of the ^1^H NMR spectra of **4** (**a**), with subsequent addition of two equiv. of Cu^2+^ (**b**) and two equiv. of EDTA (**c**) (see Figure S4 for full spectra). [4] = 2.2 mM; D_2_O:CD_3_OD 50:50; T = 298 K.

Further evidence on the coordination of Cu^2+^ to **4** was obtained through NMR experiments (Figure 4 and Figure S5). The addition of two equivalents of Cu^2+^ to sensor **4** resulted in the disappearance and/or broadening of the ^1^H NMR peaks of the aromatic region, which arise from the paramagnetic effect of Cu^2+^ ion exerted over the nearby DPA protons. Subsequent addition of ethylenediaminetetraacetate tetrasodium salt (EDTA), a stronger Cu^2+^ complexing agent, resulted in the regeneration of the aromatic ^1^H NMR signals, which indicates the removal of the metal from the DPA unit of **4**.

### 3.3. Anion Sensing Studies

**Figure 5:**
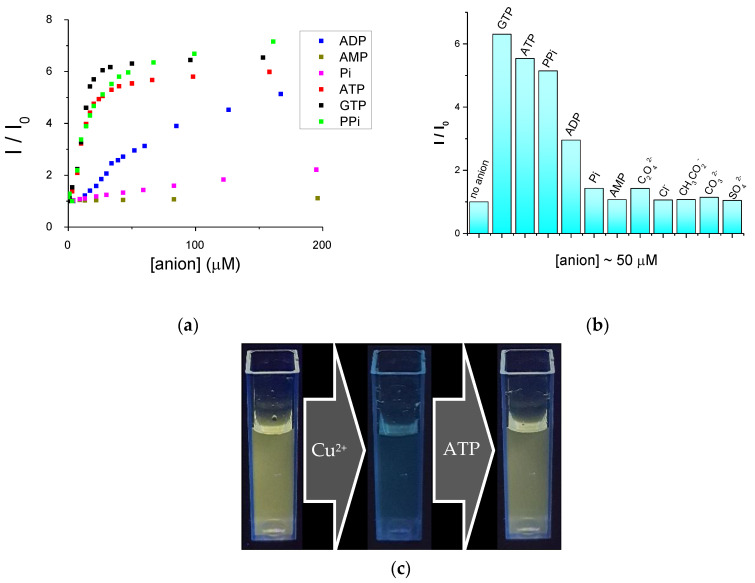
(**a**) Variation on the emission of (**4**-Cu)^2+^ upon addition of different phosphate derivatives (λ_exc_ = 458 nm); (**b**) comparison between (**4**-Cu)^2+^ in the absence (first bar) and in the presence of ~50 µM (10 equivalents) of anion; (**c**) a solution of **4** with sequential addition of Cu^2+^ and ATP, under UV light (365 nm). Conditions: [4] = 5.0 µM; pH 7.0 ± 0.2 buffered with 10 mM HEPES; T = 298 K; λ_exc_ = 458 nm.

After testing the coordination of **4** with Cu^2+^, a series of phosphate derivatives were subsequently added to (**4**-Cu)^2+^ in order to determine whether this system is suitable for the detection of anions. The results are shown in Figure 5a.

As shown, the addition of specific phosphate anions induces the recovery of the fluorescent signal from the chemosensor system. In particular, adenosine 5′-triphosphate (ATP), adenosine 5′-diphosphate (ADP), guanosine 5′-triphosphate (GTP) and pyrophosphate (PPi), which possess the highest concentration of negative charges, induce the strongest response, an effect that indicates the influence of electrostatic interactions in the sensing mechanism (Figure 5b). Inorganic phosphate (Pi), adenosine 5′-monophosphate (AMP) and several other anions induced much smaller changes to the emission of (**4**-Cu)^2+^. The fact that the fluorescent signal was recovered (Figure 5c), as well as the concentrations of anion required for this to occur, suggest that the Cu^2+^ metal ion was being displaced by the presence of the anion. These results are consistent with the fact that PPi, ATP, GTP and ADP form complexes with copper (II) with stability constants in the range of 7.5–6.0 log units, significantly higher than that found for the (**4**-Cu)^2+^ complex. The complexes of the other anions with Cu^2+^, on the other hand, display lower stability constants (about 3.3 log units) and are therefore less efficient in displacing Cu^2+^ from the sensor [30].

Another strong indication of this was when we used an excess of Cu^2+^ prior to adding anion. For example, in the case of GTP (Figure S6), the fluorescent signal only started to recover after an equivalent of anion was added, which suggests that the anion primarily binds to free Cu^2+^ ions, and thereafter, it begins to compete with **4** for the remaining Cu^2+^, ultimately resulting in its displacement from our sensor.

### 3.4. Super-Resolution Structured Illumination Microscopy (SR-SIM) Live Cell Imaging

In order to understand the viability of using chemosensor **4** for intracellular measurements, biocompatibility and live-cell super-resolution microscopy studies were performed, namely, super-resolution structured illumination microscopy (SR-SIM).

**Figure 6:**
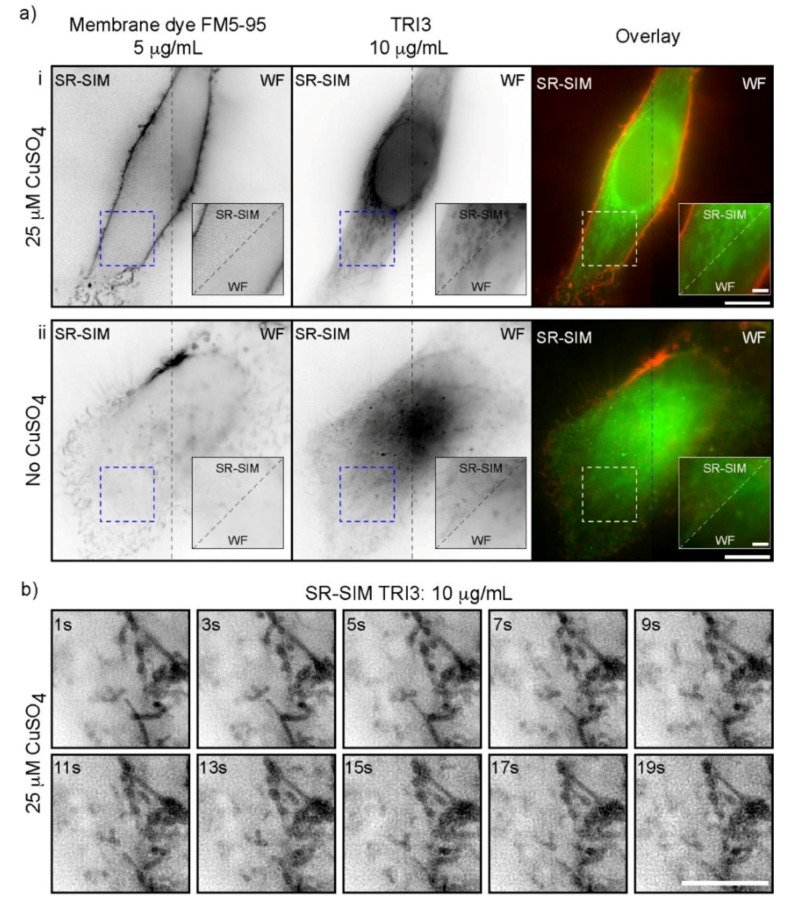
(**a**) SR-SIM and WF live-cell imaging of HeLa cells stained with 10 μg/mL of **4** and 5 μg/mL FM5-95 membrane dye (5 min in DMEM) with (i) or without (ii) 25 μM CuSO_4_. Insets are zoomed highlights of the regions defined by the dashed squares. Scale bars are 10 μm in the full-sized images and 2 μm in the insets. (**b**) SR-SIM continuous time-lapse at 1 super-resolution frame per second (20 s total imaging time) of HeLa cells stained with 10 μg/mL of **4** (for 5 min in DMEM) showing mitochondrial dynamics are not affected in the presence of **4**. Scale bars are 5 μm.

Incubation of HeLa cells with concentrations of **4** up to 12 micromolar revealed a high degree of biocompatibility, and this cell line had a survival rate comparable to the untreated cells in media with or without Cu^2+^ over a 24 h incubation period (data not shown). Microscopy experiments using SR-SIM showed high intracellular uptake of the chemosensor (Figure 6a). Microscopy experiments conducted without the addition of Cu^2+^ indicated a homogenous uptake of **4** throughout the whole intracellular space. However, in the presence of Cu^2+^ ions (approximately 2 equivalents), a clear contrast in the fluorescence was observed within cellular structures. Indeed, we observed a much stronger luminescent signal from **4** in mitochondria and cell membranes with no effect on the mitochondrial dynamics (Figure 6b).

This observation is consistent with the fact that ATP (and the product of its hydrolysis, PPi) is significantly more concentrated in mitochondria (where it is produced), and cell membranes (given its role in active transport), which ultimately leads to a recovery of the fluorescence of **4**, as opposed to the remaining intracellular space, where Cu^2+^ effectively quenches the sensor’s luminescence.

## 4. Conclusions

A new fluorescent chemosensor based on a naphthalimide unit bound to two DPA through a triazine spacer was synthesized and fully characterized. The chemosensor revealed a high selectivity towards Cu^2+^ ions, even in the presence of an excess of other divalent metal ions, and it exhibited strong quenching in the presence of Cu^2+^, with a 1:1 binding ratio.

Displacement of Cu^2+^ from the DPA centers was observed upon the subsequent addition of phosphate anion derivatives, which induced a recovery in the fluorescence of the chemosensor.

The chemosensor showed a high degree of biocompatibility, with no observable cell death after 24 h incubation. Live cell imaging studies using a human cell line and SR-SIM allowed us to obtain selective fluorescence enhancement in regions of ATP accumulation. These results can be further optimized for future work on selective mitochondrial fluorescent staining for microscopy studies.

These results highlight the potential of this fluorescent chemosensor for further biological studies on intracellular ATP accumulation.

## Supplementary Materials

The following are available online at https://www.mdpi.com/2079-6374/10/9/129/s1, Full NMR spectra and ESI-MS characterization of 4, Figure S1: UV-Vis spectra of **4** in the presence of divalent cations; Figure S2. Emission spectra of **4** in the absence and presence of 5 equivalents of metal cation. Conditions: **4** = 5.0 µM; pH 7.0 ± 0.2 buffered with 10 mM HEPES; T = 298 K; λ_exc_ = 458 nm; Figure S3: Proposed binding mode for Cu^2+^ to chemosensor 4; Figure S4: Normalized emission changes in **4** upon addition of Cu^2+^ (black dots) and respective exponential fitting for a 2:1 binding model (red line). Conditions: **4** = 5.0 µM; pH 7.0 ± 0.2 buffered with 10 mM HEPES; T = 298 K; λ_exc_ = 458 nm; Figure S5: ^1^H NMR spectra of **4** (blue), with subsequent addition of two equivalents of Cu^2+^ (green) and two equivalents of EDTA (red). Spectra were acquired in a 1:1 mixture of CD_3_OD and D_2_O; Figure S6: Normalized intensity from a solution containing **4** (5 µM) and Cu^2+^ (25 µM) upon increasing concentrations of GTP. Conditions: **4** = 5.0 µM; pH 7.0 ± 0.2 buffered with 10 mM HEPES; T = 298 K; λ_exc_ = 458 nm.
