# Supplementary material for: Selective Coordination of Cu2+ and Subsequent Anion Detection Based on a Naphthalimide-Triazine-(DPA)2 Chemosensor
Source: Biosensors (Basel). 2020 Sep 22;10(9):129. doi: 10.3390/bios10090129 (PMC7558417; doi:10.3390/bios10090129)
Supplement: Supplementary file 1 [file biosensors-10-00129-s001.docx]

**Supporting Information**

**Selective coordination of Cu^2+^ and subsequent anion detection based on a naphthalimide-triazine-(DPA)_2_ chemosensor**

Miguel Santos,*^a^* Mani Outis,*^a^* Pedro M. Pereira,*^b^* Pedro Mateus,*^a^* Artur J. Moro,*^a^**

*^a^* *LAQV-REQUIMTE, Departamento de Química, CQFB, Universidade Nova de Lisboa, Monte de Caparica, Portugal. E-mail:ajm12769@fct.unl.pt*

*^b^ Bacterial Cell Biology, MOSTMICRO, Instituto de Tecnologia Química e Biológica António Xavier, Universidade Nova de Lisboa, Oeiras, Portugal*

*NMR characterization of* ***4*** *(DMSO-d_6_)****:***

**^1^H NMR**

**^13^C NMR**

**^1^H-^1^H COSY NMR**

**^1^H-^13^C HSQC NMR**

**^1^H-^13^C HMBC NMR**

*High resolution electrospray ionization mass spectrum of* ***4*** *(positive mode)*


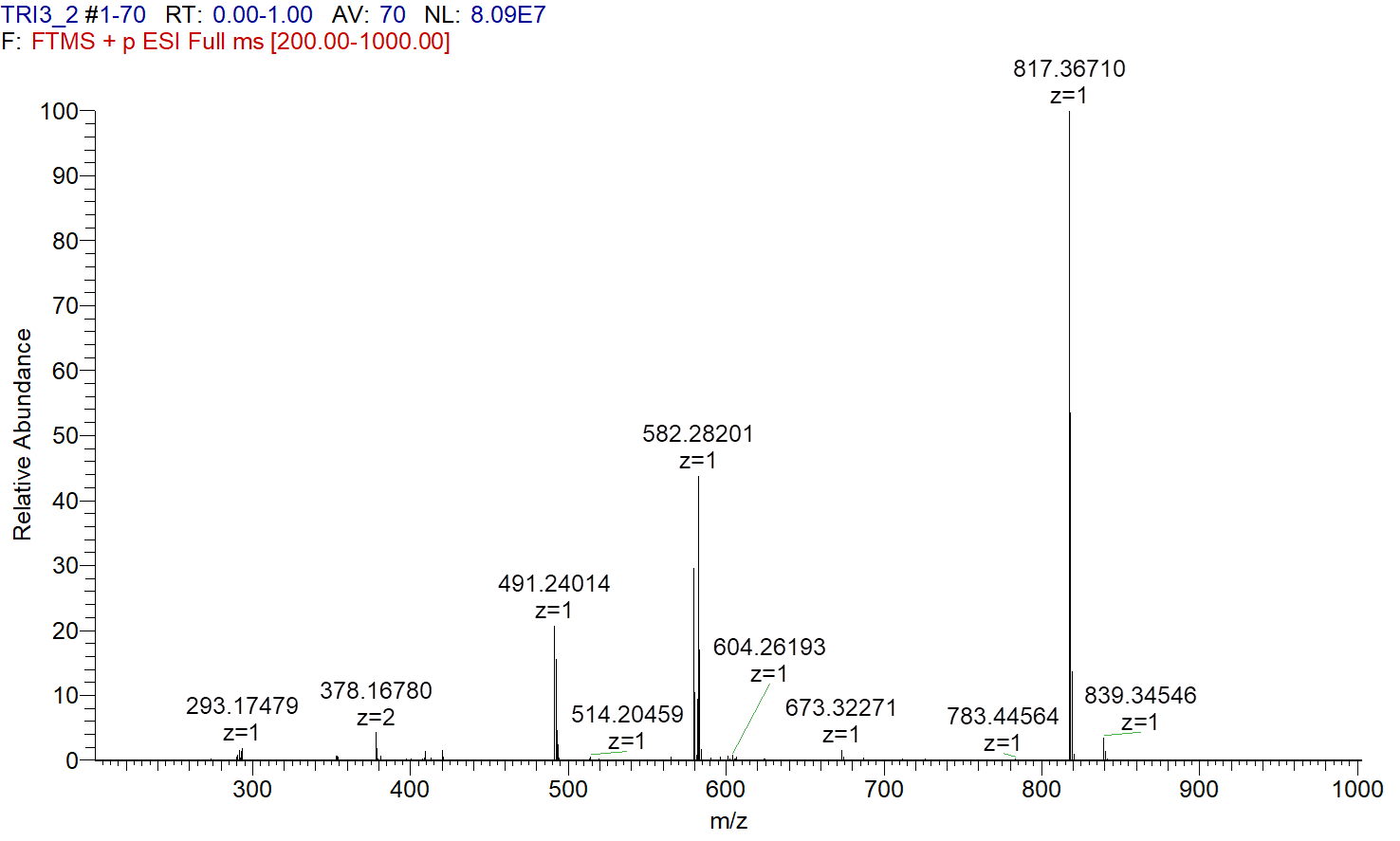

Figure S1. UV-Vis spectra of **4** in the presence of divalent cations.

Figure S2. Emission spectra of **4** in the absence and presence of 5 equivalents of metal cation. Conditions: [**4**] = 5.0 µM; pH 7.0±0.2 buffered with 10 mM HEPES; T = 298 K; λ_exc_ = 458 nm.


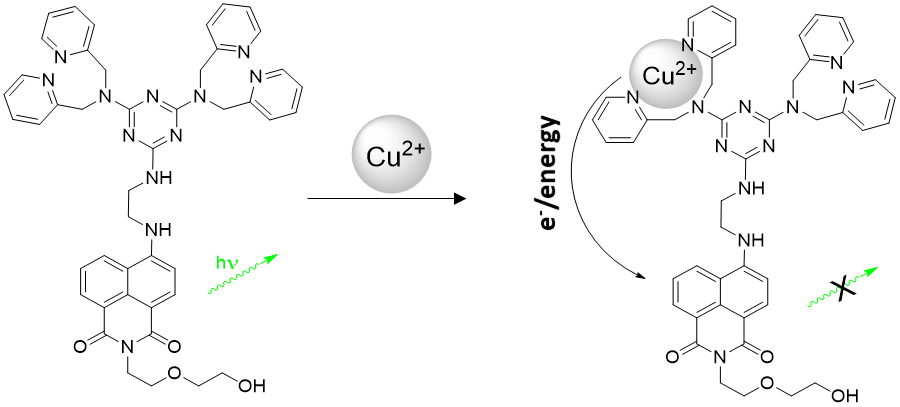


Figure S3. Proposed binding mode for Cu^2+^ to chemosensor **4**.

Figure S4. Normalized emission changes in **4** upon addition of Cu^2+^ (black dots) and respective exponential fitting for a 2:1 binding model (red line). Conditions: [**4**] = 5.0 µM; pH 7.0±0.2 buffered with 10 mM HEPES; T = 298 K; λ_exc_ = 458 nm.

Figure S5. ^1^H NMR spectra of **4** (blue), with subsequent addition of two equivalents of Cu^2+^ (green) and two equivalents of EDTA (red). Spectra were acquired in a 1:1 mixture of CD_3_OD and D_2_O.

Figure S6. Normalized intensity from a solution containing **4** (5 µM) and Cu^2+^ (25 µM) upon increasing concentrations of GTP. Conditions: [**4**] = 5.0 µM; pH 7.0±0.2 buffered with 10 mM HEPES; T = 298 K; λ_exc_ = 458 nm.
